# Lessons learnt from implementing blended ‘integrated’ learning into an undergraduate medical curriculum

**DOI:** 10.15694/mep.2017.000129

**Published:** 2017-07-17

**Authors:** Shashidhar Venaktesh Murthy, Torres Woolley, Yeshwanth Rao K, Nagaraja Haleagrahara, Bunmi Malau-Aduli

**Affiliations:** 1 James Cook University; 2 Manipal University

**Keywords:** Blended Learning

## Abstract

This article was migrated. The article was marked as recommended.

**Method:**This study utilized a mixed-method design involving a cross-sectional survey (n=111, response rate=61%) to compare Year 2 medical student perceptions of content delivered by a Blended Integrated Learning (BIL) approach versus a traditional didactic teaching (TT) approach, plus 2 focus groups to explore learner perceptions of the BIL approach and brainstorm improvements.

**Results:** Most medical students preferred the BIL approach over TT with respect to ‘practically applying basic sciences to a patient case’ and ‘knowledge retention in the subject’ (53% versus 30%, and 51% versus 35%, respectively). However, most medical students preferred TT with respect to ‘level of interaction with other students and lecturers while reviewing teaching materials’ (78% versus 11%), ‘overall enjoyment of learning’ (54% versus 32%), and ‘understanding the lecture content faster’ (49% versus 39%). Focus groups identified what did and did not work with the BIL approach and brain-stormed specific improvement strategies.

**Conclusions:** Students preferred BIL over TT for knowledge retention and integrating basic sciences into common clinical cases, but found BIL less preferable for aspects related to learning engagement. However, focus groups identified a variety of strategies to promote student engagement in BIL by improving online content, delivery processes and further innovative use of technology.

## Introduction

The undergraduate entry Bachelor of Medicine, Bachelor of Surgery (MBBS) James Cook University (JCU) medical school was established in North Queensland in the year 2000). ^1^ Since inception, the first three pre-clinical years of the JCU medical curriculum have provided a systems-based introduction to the foundations of medicine, requiring students to enrol in two chained subjects for each academic year with 4-5 modules in each subject. However, while a key focus of the curriculum, both horizontal integration of the basic science modules and vertical integration between basic sciences and the later clinical content has often proved difficult to implement. Furthermore, the JCU medical school has experienced reduced teaching staff to student ratios over the past several years. As a result, there has been much recent review and discussion around the future delivery of content material at the JCU medical school.

Technology-enhanced interactive learning (also known as e-learning) is one method JCU medical educators have considered using to assist the delivery of basic sciences information via online resources. E-learning can allow student learning to be individualised (adaptive learning), enhance learners’ interaction with each other (collaborative learning), and realign the educator’s role from disseminator to facilitator of the learning process. ^2, 3^

However, the literature shows e-learning approaches to have had mixed success. Some studies show e-learning approaches to be associated with increased educator and learner satisfaction, instructional richness, ease of use and access to content material, interactivity, and cost effectiveness. ^4- 6^ Other studies show e-learning approaches to be associated with decreased social interaction during learning activities, and low learner satisfaction and low adoption rates as a result of technical IT and course design issues and leaners’ level of technological capability. ^7- 9^ In medical education, this confusion is compounded by a lack of investigation into the effectiveness of e-learning approaches in the field. ^10^

One of the more promising e-learning approaches is Blended Learning which combines online digital media with traditional classroom methods. Blended Learning requires the physical presence of both teacher and student, with some element of student control over time, place, path, or pace. ^11^ A major advantage of Blended Learning to the JCU medical school is that it has the capacity to allow integration of a range of basic sciences information into common clinical case scenarios - facilitating horizontal integration of the basic science modules in Years 1-3 and also vertical integration between basic sciences and the later clinical content in Year 4-6 of the medical curriculum.

The importance of integrating basic sciences with clinical cases in order to reduce loss of basic science knowledge when medical students transition from pre-clinical to clinical years has been well documented. ^12- 15^ In addition, integration of basic science and clinical concepts throughout the curriculum helps students develop clinical reasoning skills, ^16- 17^ fosters knowledge retention, and reinforces the relevance and application of basic sciences to clinical context. ^18- 19^

In 2016, the JCU medical school implemented a Blended Learning approach into a week’s teaching content around haemostatic disorders for the 2 ^nd^ Year module ‘Haematology and Renal Medicine’ (HRM). The ‘haemostatic disorders’ week adapted the standard Blended Learning approach by presenting clinical cases using interactive videos and questions to emphasise the underlying basic and clinical science concepts and their relevance - taking a Blended ‘Integrated’ Learning’ (BIL) approach. The integrated content was developed by a multi-disciplinary team of four experts (Physiologist, Pathologist, Pharmacologist and Clinician). The remainder of content for the 13-week HRM module was delivered via a more traditional, didactic teaching approach.

This study compared Year 2 medical student perceptions of the introduced Blended Integrated Learning (BIL) approach used to deliver the third weeks teaching content on ‘haemostatic disorders’ versus the didactic (traditional) teaching approach to deliver the second weeks teaching content on a related pathophysiology topic ‘red blood cell disorders’.

## Methodology

### Setting and study design

This study utilized a mixed-method design involving a cross-sectional survey and two focus groups. The survey and focus groups were all conducted at the JCU medical school during first semester of the 2016 academic year. The study assessed medical students’ perceptions of the BIL approach versus a more TT approach. The survey was conducted immediately after the ‘BIL’ week focussing on haemostatic disorders, which followed the traditional teaching week focussed on RBC disorders, while the focus groups were conducted approximately four weeks after the survey. Ethical approval was obtained from the JCU Human Ethics Committee [H5595].

### Description of the BIL teaching approach

Year 2 teaching staff from Physiology, Pathology and Pharmacology disciplines co-developed content material for a week of teaching in the JCU Blackboard learning platform. This integrated and adaptive blended learning (BIL) mode of teaching was embedded in the pre-clinical Year 2 module ‘Haematology and Renal Medicine’ (HRM) to deliver the third week’s topic of ‘haemostatic disorders’. Currently at the JCU medical school, traditional teaching (TT) is mainly delivered didactically and via small group activities, with clinical case integration not being a necessary component. For example, the TT week topic ‘red blood cell (RBC) disorders” was delivered by a Pathologist. In contrast, the BIL teaching for the topic ‘haemostatic disorders’ was presented by the teaching staff utilizing interactive videos and questions embedded into practical clinical scenarios to emphasise the underlying basic and clinical science concepts with an emphasis on their clinical relevance. For the BIL approach, content was developed by a multi-disciplinary team involving a Physiologist, Pathologist, Pharmacologist and a Clinician. The details of both methods are as follows.

Summary of the ‘Traditional Teaching’ (TT) method:

• Overview document containing weekly learning objectives and teaching sessions uploaded in blackboard learning management system two days prior to the commencement of the week
• The week starts with an introductory lecture of one hour duration
• A three hour guided Learning Session (GLS) delivered on the 2 ^nd^/3 ^rd^ day which can be a didactic lecture plus a small group activity or laboratory activity
• The last teaching session of the week is a one hour synthesis session wherein questions and answers are discussed in the classroom

Summary of the ‘BIL’ method:

• Overview document containing weekly learning objectives and teaching sessions uploaded in blackboard learning management system two days prior to the commencement of the week
• The week starts with an introductory lecture covering weekly learning objectives built around two clinical scenarios
• Following introductory session, multiple short lecture video segments involving clinical cases and related basic science lectures with built-in quizzes using Techsmiths Camtasia Studio and Camtasia for Windows are released online (total 60-80 minutes) covering an Introduction, Physiology, Pathology and Pharmacology related to the clinical scenarios
• One hour presentation of clinical cases by a clinician delivered on the 2 ^nd^/3 ^rd^ day
• The last teaching session of the week is a one hour synthesis session wherein clinicians presented patient cases in the classroom. The session was completed with a review session to confirm students’ understanding of key concepts, whereby questions are asked via a built-in student response system (Keepad from TurningPoint) and then answers discussed in the classroom.

### Participants and data collection

The study was conducted with the JCU Year 2 medical student cohort (183 students). All Year 2 students were exposed to both the BIL and traditional teaching (TT) weeks in weeks 1 (TT method) and 3 (BIL method). In week 3, all students present in the Synthesizing Session were invited to complete a de-identified survey comparing their experiences with the BIL teaching method to the TT teaching method. To ensure power balance, the survey instrument was administered by the researchers not involved in teaching and assessing the students.

### Survey questions

The first 8 survey questions asked students to rate both the BIL approach and the TT approach on a Visual Analogue Scale (VAS) across each item from ‘not at all’ to ‘excellent’. The first 8 survey questions are described in Table 1. The 9 ^th^ question asked students to score their preferred learning style on a VAS from ‘traditional didactic learning’ to ‘integrated, case-based learning’. In addition, the survey also contained 2 open-ended survey questions on “Were there any significant learning advantages OR disadvantages to the BIL teaching week?”, and “Any suggestions for improving the BIL teaching week?”

A Visual Analogue Scale (VAS) is a psychometric response scale used to quantify subjective characteristics or attitudes that are believed to range across a continuum of values and cannot be directly measured. There is evidence showing that a VAS has superior metrical characteristics than discrete scales, thus allowing a wider range of statistical methods to be applied to the measurements. ^20^ Following standard practice, the VAS used in this study was a horizontal line, 100 mm in length, anchored by word descriptors at each end. The VAS score is determined by measuring in millimetres from the left hand end of the line to the point that the respondent marks. Figure 1 gives an example of the VAS used in this study to inform the Year 2 medical students how to rate the BIL and TT approaches across the first 8 survey items.

### Focus group data

Focus groups (FGs) were used to explore students’ experiences and perceptions of the BIL method and also brainstorm how the BIL approach could be improved for future cohorts. The questions asked in the FGs were developed after content analysis of data collected in the 2 open-ended survey questions (key learning advantages or disadvantages to the BIL teaching week, and suggestions for improving the BIL teaching week), and explored the major themes identified in more detail. The interviews were conducted by the author TW, who had no formal teaching role with the students. Students were recruited to the FGs by a question in the survey asking if they were “willing to participate in a discussion group with 5-6 other students (lunch provided)” by providing their email address in a boxed section.

### Data analysis

Survey data for the 9 VAS items were coded numerically (score between 0-100) and entered into the computerized statistical package for social sciences (SPSS) release 20 for Windows. To compare student preferences for the traditional teaching (TT) approach versus the BIL approach across the first 8 survey questions, student scores were categorized into the BIL approach being rated either ‘better’, ‘the same’ (<5 mm difference between student scores on the two VAS) or ‘worse’ than the TT approach ( Table 1).

For the qualitative data analysis, the thematic analysis undertaken in this study followed a grounded theory approach by inductively extracting themes from participant responses, and then developing these themes into theory by identifying the organizing concepts which linked themes. ^21^ Membership of the two focus groups comprised seven Year 2 students, with females slightly over-represented (4F:3M); although about 65% of students in all cohorts are female. As participants were all Year 2 students whom had experienced both BIL and TT approaches, they can be considered to be information-rich cases that allowed the research questions to be adequately explored. ^22^

Data analysis involved listening to the electronic transcripts of both groups repeatedly, using immersion to develop a high level of familiarity with the data, and then manually coding the data into separate summary concepts. As the analysis was focussed on understanding the key problems with the BIL approach identified in the survey, and then to brainstorm solutions to these problems, transcripts were not electronically transcribed, nor were additional FGs organized to reach “theory-saturation point”. ^22^

The 3 main areas covered in the FGs were: what students found good about the BIL week with the online case-based videos, quizzes and slides (focusing on flexibility of use, the video quizzes, integrated slides, knowledge retention, whether it made content easier to understand, and to think more clinically), what students find good about the BIL week (focusing on level of interaction with peers and lecturers, and issues with learning at home via online resources), and how could BIL online resources be improved with regards functionality, enjoyment, efficiency, effectiveness/quality, and being more compatible for students who prefer a paper-based and/or lecture-based learning style.

## Results

### Descriptive statistics

A total of 111 JCU Year 2 medical students responded to the survey from a possible 182 (response rate = 61%). Most medical students preferred the BIL approach over the TT approach with respect to ‘assisting you to practically apply basic sciences to a patient case’ and ‘assisting your knowledge retention in the Haematology subject’ (53% versus 30%, and 51% versus 35%, respectively).

In contrast, most medical students preferred the TT approach over the BIL approach with respect to ‘assisting in your level of interaction/collaboration with other students and teachers while reviewing the materials’ (78% versus 11%), ‘your overall enjoyment of learning Haematology’ (54% versus 32%), ‘enabling you to learn and understand the lecture content faster’ (49% versus 39%), and ‘helping you learn what you need for this subject and about Haematology in general’ (44% versus 37%). Students reported the BIL and TT approaches to be roughly similar with respect to ‘assisting your deeper understanding of the Haematology subject’ (TT = 39%, BIL = 37%), and ‘the overall quality of the learning experiences’ (TT = 42%, BIL = 38%). Overall, 56 (51%) of Year 2 students preferred traditional didactic learning, while 53 (49%) preferred the integrated, case-based learning used in BIL.

### Focus group (FG) data

The two FGs identified a number of benefits as well as disadvantages to the BIL approach. The most commonly reported benefit was the extra flexibility for study afforded by the BIL videos. Videos and audio assisted students in being able to review all the content at their own pace by pausing or slowing the video, and being able to do so at a time that is best for them. Typical student comments included:

*“Could go through content at my own pace in a quiet environment that was conducive to learning”*;

*“It was quite good because I could listen to information at my own pace, be tested [by quizzes], and search up unknown terms then and there”;*

*“I enjoyed it because I could SLOW [lecturer] down to ¾ speed and really absorb what he was saying.”*

*“I liked being able to pause and re-wind the videos, as I was able to work at my own pace. I found this so much more effective than traditional teaching as I constantly feel lost in normal lectures if I do not write out the content beforehand”*

*“Liked being able to go look & listen to audio with visuals allowed me to consolidate and learn more effectively - I really like this approach”.*

However, as the BIL videos delivered content predominately by audio linked to slides, this process was considered slower and sometimes less effective for some learners:

*“Increased time to do BIL - took me 4 hours - but deeper learning, so worth it”;*

*“I had to complete the videos which took much longer than the quoted “70 minutes” due to stopping/starting for taking notes”*

*“Easier to dis-engage if I’m just looking at slides”;*

*“It is easier to get distracted when you’re just watching videos”;*

*“The video/audio recordings are helpful, but I think the slides need more detail as I like to refer back to notes, not recordings. I don’t feel like I retain as much from just hearing the content - we need more detailed notes”*

*“It means there are a lot more lecture-style teaching which can become tedious - it took longer to complete the material”*

However, some of these students thought they would get faster at learning via this approach with more experience, while other students did not think they would:

*“I have a personal preference for traditional learning, although I can get used to the idea of BIL over time - it seems like a good method of teaching”;*

*“Although I gained a lot of benefits from BIL, I can understand that some students will have a bit of difficulty in adapting to the new learning style. I really like BIL because it encourages active learning, and I understand the week’s material faster than I would in traditional learning”,*

*“I am most likely not going to watch the videos outside of class; therefore, I prefer the traditional method”;*

*“I feel very disadvantaged as my learning style is seeing a lecturer talk, and use their hands to explain, and just being present. I was disengaged and feel I don’t understand the content for this week”*

Videos with the embedded quizzes were also very popular, as it helped engage the students, assisted them in becoming more involved in active learning, improved recall, and check their understanding of words and key concepts:

*“The in-built quizzes are fantastic because you absolutely need to be listening and present to answer them”;*

*“The quiz method was very effective as it tested my understanding of content without being able to refer to notes - that is, it forced me to recall information quickly, similar to an exam situation”;*

*“The quiz questions were good in checking understanding - if I got the question wrong I was able to go back in the video and re-listen”*

*“The quizzes during the video ensured that you were actually engaging in the content being delivered”*

However, while nearly all students thought the embedded quizzes were good, students still needed interactions with lecturers and peers to bounce ideas off, ask questions, and get feedback questions around why the quiz Qs were right or wrong:

*“We had to get the concepts ourselves in isolation”;*

*“The major disadvantage was that it took away time to speak to the lecturer or tutor about questions, like in the normal GLS, which I have always found quite beneficial”;*

*“HRM is too complex a subject to not be able to engage with our lecturers and GLS tutors”*

*“Meant that I couldn’t ask tutors questions of go to a specific room with other students and discuss the GLS”*

The case-based approach allows students to “actually study medicine, not just sciences”; in particular by practically applying anatomy, physiology, pathophysiology, pharmacology etc to patient cases via a differential diagnosis approach:

*“You could learn the content by exploring the physiology, pathophysiology and treatment separately.”*

*“[BIL was] excellent for cases - I would always use a program like this for cases. As long as it is made clear how much content we need to know, then BIL is fine”*

*“Clinical scenarios were enjoyable to relate real-life application”*

*“Integration of videos with case helps develop clinical thinking. CASE BASED is SO MUCH MORE BENEFICIAL”*

*“Gave a wider range of content integrating different areas involved with clinical treatment”*

However, as this was the first time they experienced clinical reasoning, some students found it difficult to integrate all the information involved with the clinical cases, while some liked the approach but preferred it to be a little more explicit with a simpler, step-by-step description of the clinical reasoning process:

*“I think BIL would work best for theory/procedural concepts; e.g., process of hemostasis or information that is more facts based. With clinical cases, it becomes difficult to integrate the information”*

*“Bring it more back to the basics. I don’t know what I’m seeing with a low haemoglobin. I would like the Lecturer to dissect the case - walk us through it. It could be this, but it’s not because..”*

Suggestions for improving the BIL approach were also brainstormed in the two FGs. Suggested improvements for the videos, quizzes and online resources included:

• More quizzes
• Ensure high quality of the audio and lecture slides
• improvements needed to make the audio more stimulating; such as changes in modulation “seems unnatural - scripted”, be louder, and include jokes,
• the lecturer needs to be more engaging on the videos; suggestions include showing the lecturer with mouth moving or talking in the videos (even if just a quivering mouth on a photo of the Lecturer)
• Video the Lecturer talking and drawing diagrams on a Whiteboard to walk people thru key concepts
• All online slides should be on a single document, and videos should be kept to a minimum by combining them into longer videos
• Videos need to be uploaded by the weekend before the SS to allow students more time to view them and prepare questions for the Lecturer
• Have a weekly overview document/clearer direction to guide study

Suggested improvements for weekly Synthesizing Session (SS) included:

• Extend length of SS to allow a comprehensive discussion and feedback session
• Quiz answers should be released during the SS (“We don’t want to learn the wrong thing from what you’ve written - got to have the answers”)
• Livestream the SS discussion for greater flexibility - students in the SS class or at home could watch the Livestream online, allowing students to text questions anonymously to the Lecturer if at home sick or if a more ‘shyer’ student in class
• Video and simultaneously live-stream the SS

## Discussion

The literature suggests this is the first study to have evaluated the effectiveness of a blended and integrated learning approach in pre-clinical undergraduate medical education. The survey and focus group findings found that the BIL approach used to deliver a week’s content in the Year 2 ‘Haematology and Renal Medicine’ module allows students very flexible access to the lecture content material at their own pace and in their own time and location. In addition, the survey findings suggests the BIL approach is more effective than traditional didactic teaching for integrating basic sciences into a patient case, and assisting students’ knowledge retention. Thus, these findings suggest that a BIL approach has much potential in undergraduate medical education, where flexible learning is becoming more desired by the student body, coupled with the necessity in medicine for students to move away from simply learning facts, and start making connections and long-term understandings about how the basic science component function as part of the clinical decision-making process for the differential diagnosis of clinical cases.

However, the survey found Year 2 students also thought the BIL approach was worse than TT in allowing interactions with other students and lecturers while reviewing teaching materials. This is perhaps the biggest problem with the BIL approach in its original form, as social interactions and collaborative learning are known to affect the perceived usefulness of blended learning. ^8- 9, 23^ Interactive instruction actively engages the learner, and encourages the learner to construct and produce knowledge in meaningful ways, while students also teach others interactively and interact generatively with their lecturer and peers - these factors allow for co-construction of knowledge which promotes engaged learning that is problem- and outcome-based. Reduced opportunities for interaction means reduced opportunity to ask questions about content that students find confusing. Lack of interaction is known to result in learner frustration and a sense of isolation ^24^ as well as increasing the likelihood of learners disengaging and/or misinterpreting key concepts, ^25^while face-to-face interactions provide enjoyable social communications. This study shows that face-to-face interaction is viewed as highly important by medical students, even in e-learning spaces, to provide sense of community and ensure that effective learning has occurred.

As a result, much time in the focus group discussions were spent brainstorming ways of how more interaction could be built into the medical school’s BIL approach. Preferred solutions include extending the length of the SS to allow a comprehensive discussion and feedback session, a variety of strategies to make the audio more stimulating and the visuals more engaging, the online resources delivered in a more streamlined package, and further use of technology to make the BIL approach increasingly more versatile.

While the BIL approach also was perceived less favourably than TT with respect to enjoyment of learning and the speed of which students learnt and understood key concepts, much of this was a result of resources and processes requiring fine-tuning that only hindsight and evaluation of the student experience can bring. It has been well documented that environmental factors such as system functionality affect the perceived usefulness of blended learning approaches. ^8, 23^ The issues with the JCU CMD’s BIL approach are somewhat understandable given this was the first time the medical school had delivered multi-disciplinary content to the pre-clinical students using online videos embedded with quizzes that also integrated basic science information into practical clinical cases. In particular, the skills required to create and deliver technology-related materials differ from those needed for creating and delivering content via traditional teaching. Lastly, the study participants were Year 2 medical students, whom have not previously been exposed in the JCU curriculum to either the use of blended online resources, or lecture content that integrates basic sciences into clinical case to show the reasoning leading to a differential diagnosis for common clinical cases.

Therefore, this study identifies the need for adequate, regular staff training to enhance the BIL experience for students and their instructors, as well as rigorous, quality-improvement focussed evaluations of Blended Learning resources and delivery strategies; especially if this approach involves student users less-experienced with online delivery methods. Overall, our study adds further confirmation that e-learning is perceived by learners as a complement rather than as a substitute for traditional educator-led teaching methods. ^5^ However, our study also suggests that with well-evaluated e-learning approaches, online technologies may be able to better facilitate engagement of students and deliver integrated content in ways that would be difficult to achieve using traditional didactic teaching methods.

## Limitations

While the nature of the JCU MBBS program is not representative of all medical schools across Australia or the world, some commonality is expected between the experiences of Year 2 JCU medical students with students in other medical courses regarding integrating basic science knowledge into clinical scenarios. However, it is possible that obtaining student perspectives from the other pre-clinical years (1 and 3) may elicit different experiences. Thus, the study’s major limitation is likely to be the survey response rate (61%), which while not unreasonable, does allow the potential for non-respondent bias; though comparison of the respondents’ profile to the overall Year 2 cohort showed they were representative across age and gender.

Another limitation is that the described BIL approach does not have a precedent in Medicine, and while based on a sound educational theory, all the resources were developed de novo by staff of the JCU medical school. However, the mixed-methods approach used in this study would allow for some triangulation of data to increase the rigour and validity of the research. A further limitation may be the use of the VAS method. The VAS method may also be considered subjective in this study, as these scales are of most value when looking at change within individuals, and are of less value for comparing across a group of individuals at one time point; thus, some caution is required in reporting this data. However, the VAS was used in this study in a comparison approach, which would somewhat negate this problem, as well as potential biases with using non-validated question items in the survey.

## Conclusions

Medical students preferred the ‘blended, integrated learning’ (BIL) approach using online resources over traditional educator-led classroom teaching in the key areas of integrating basic sciences knowledge into common clinical case scenarios, and knowledge retention. However, BIL was considered less preferable for enjoyment of learning, speed of learning, understanding the content, and enhancing interactions with peers and teachers - all important aspects of promoting engagement in learning. However, the focus group discussions led to a range of improvements to be implemented for the BIL approach in the 2017 teaching year that should address these main problems identified by the students.

While our study adds further confirmation that e-learning is preferred by most students as a complement to, rather than as a substitute for, traditional educator-led teaching methods, online technologies may be able to facilitate student engagement and deliver integrated content in ways that would be difficult to achieve using traditional didactic teaching methods. Finally, the study suggests teachers less experienced in blended learning approaches need to rigorously evaluate their newly-developed e-learning resources and their delivery processes with a quality-improvement focus, as creating and providing appropriate web-based educational content is very different from creating and providing content via traditional teaching methods.

**Table 1.** JCU Year 2 medical student perceptions of Blended Integrated Learning (BIL) versus Traditional Teaching (TT) methods: which approach is better or worse?

Was the Blended Integrated Learning (BIL) approach better than Traditional Teaching for ...	Worse	About the same	Better
... your overall enjoyment of learning Haematology?	59 (54%)	16 (25%)	35 (32%)
... the overall quality of the learning experiences?	46 (42%)	22 (20%)	42 (38%)
... helping you learn what you need for this subject and about Haematology in general?	48 (44%)	21 (19%)	41 (37%)
... enabling you to learn and understand the lecture content faster?	52 (49%)	13 (12%)	42 (39%)
... assisting your deeper understanding of the Haematology subject?	42 (39%)	26 (63%)	40 (37%)
... assisting your knowledge retention in the Haematology subject?	38 (35%)	16 (15%)	55 (51%)
... assisting you to practically apply basic sciences to a patient case?	33 (30%)	18 (17%)	58 (53%)
... assisting in your level of interaction/collaboration with other students and teachers while reviewing the materials?	84 (78%)	12 (11%)	12 (11%)

**Figure:**
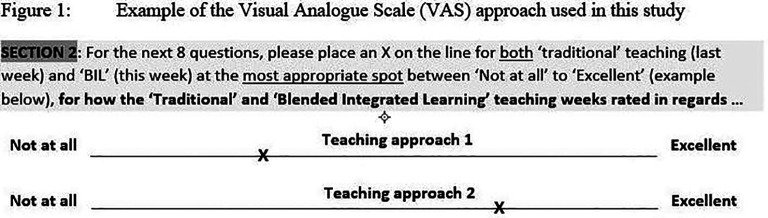


## Take Home Messages

• Medical students preferred the ‘blended, integrated learning’ approach using online resources over traditional educator-led classroom teaching for ‘knowledge retention’ and ‘integrating basic sciences knowledge into common clinical case scenarios’
• Students preferred traditional educator-led classroom teaching for aspects related to learning engagement: level of interaction with other students and lecturers while reviewing teaching materials, overall enjoyment of learning, and understanding the lecture content faster
• Online technologies can better facilitate engagement of students and deliver integrated content in ways that would be difficult to achieve using traditional didactic teaching
• Confirms e-learning is best used to complement traditional educator-led teaching methods rather than as a substitute
• Teachers less experienced in blended learning approaches need to rigorously evaluate their newly developed e-learning resources and their delivery processes for quality improvement purposes, as creating and providing appropriate web-based content is very different from creating and providing content via traditional teaching methods

## Notes On Contributors

Dr Torres Woolley (PhD) is the Evaluation Coordinator for the JCU College of Medicine & Dentistry in Townsville. His work predominantly involves evaluation of graduate outcomes and curriculum learning activities across the 6 undergraduate years.

Dr Shashidhar Venaktesh Murthy (MD) is the Associate Professor for Pathology at the JCU College of Medicine & Dentistry, Townsville, Australia. In addition to undergraduate Pathology teaching, he is interested in Digital Pathology, Educational Technology, Blended Learning and Social media to enhance student learning.

Dr Nagaraja Haleagrahara (NH) is the Senior Lecturer of Physiology at the JCU College of Public Health, Medical and Veterinary Sciences. Apart from undergraduate teaching, his interest is in Medical Education and Learning Technologies.

Dr Bunmi Malau-Aduli (PhD) is a Senior Lecturer in Medical Education and the Academic Lead for Assessment and Evaluation at the College of Medicine & Dentistry, James Cook University.

Dr Yeshwanth Rao K (MD) is the Professor of Pharmacology in Melaka Manipal Medical College (MMMC), Manipal University, Manipal, INDIA. Apart from undergraduate teaching, he is also actively involved in Medical education and Clinical trials. Other special interest includes the field of ‘technology in education.
