# Supplementary material for: Lessons learnt from implementing blended ‘integrated’ learning into an undergraduate medical curriculum
Source: MedEdPublish (2016). 2017 Jul 17;6:129. [Version 1] doi: 10.15694/mep.2017.000129 (PMC10885279; doi:10.15694/mep.2017.000129)
Supplement: Peer review history

# Reviewer report: Reviewer response for version 1

**DOI:** 10.21956/mep.19301.r28135

**Published:** 2017-08-03

**Authors:** P Ravi Shankar

**Affiliations:** 1 American International Medical University

This review has been migrated. The reviewer awarded 4 stars out of 5 I enjoyed reading this article about blended learning in an Australian medical school. I also liked the fact that a medical educator from a medical school in India was a coauthor. As a medical educator in a Caribbean medical school I examined this paper from a developing country perspective. The widespread availability of online resources makes blended and online learning more feasible for educators and students in developing countries also. I am of the opinion that the summaries of the two methods provided in the article provides insights into the detailed planning process which went into both methods. The fact that faculty members from a variety of disciplines worked together to create the module was of interest. Over the last ten years I have spend a lot of my time and efforts in both Nepal and the Caribbean in getting educators to work together. I have had mixed results. In the Main body, Introduction section, line 5: What exactly do the authors mean by ‘chained subjects’?Using clinical cases to underpin learning in the basic sciences and demonstrate the clinical relevance and utility of these subjects is becoming increasingly common in medical education. The FGDs did provide dense information though they were not conducted in the traditional manner till data saturation. I do agree with the reviewer, Dr Masters that blended learning approaches are becoming increasingly common and have been described in the literature. I do agree with Dr Masters that no educational theory has been described in the paper. It will be interesting to know the theory underpinning the blended learning initiative. The reviewer, Dr Eaton describes an important point about self-regulated learning skills which are becoming increasingly important in health education. This is a well-written paper and adds to the body of literature on this subject.

Reviewer Expertise:

NA

No decision status is available

# Reviewer report: Reviewer response for version 1

**DOI:** 10.21956/mep.19301.r28134

**Published:** 2017-08-02

**Authors:** Deborah Murdoch Eaton

**Affiliations:** 1 University of Sheffield

This review has been migrated. The reviewer awarded 4 stars out of 5 The authors undertook a carefully thought through evaluation of student perceptions on two different models of curricular content delivery – blended learning vs traditional didactic teaching.. This was from the perspectives of their preference rather than the impact on their learning, or evidencing which had better outcomes in terms of assessed knowledge or application in practice. Student preferences are important as this will impact upon engagement with taught components – and educators may then anticipate (hope?) for enhanced impact upon learning. In this study all students were exposed to both types of curricular delivery method – week 1 being tradition and week 3 being BIL – does this mean that having had a “good grounding” in week 1 through the traditional teaching methods, this would make them more receptive or able to utilise the blended learning resources better? The results would indicate that these students preferred the BIL to enhanced their skills in application to clinical cases – as one might expect, and the traditional approach (as in this design given in week 1) for “deeper understanding of the … subject” ie implication around the underpinning theoretical knowledge. Time involved in “doing it yourself” was also identified as a potential issue – again as expected (as we all look for how can we do this quickly) although the FG did acknowledge this resulted in deeper learning. All of this is useful to think through and identifies and illustrates that the core underpinning theme is that of developing SRL (self regulating learning) skills early in students – so that they can develop an ability to select the learning for their current level of skills and competence. And the embedding of self- assessment (tests) throughout allowing the learner to identify what further learning they need to do essential.This demonstrates a very nice model of using a variety of teaching techniques reflecting the range of learners likely within a course and the potential to enhance not only their specific topic expertise, but also their skills in SRL.

Reviewer Expertise:

NA

No decision status is available

# Reviewer report: Reviewer response for version 1

**DOI:** 10.21956/mep.19301.r28133

**Published:** 2017-07-17

**Authors:** Ken Masters

**Affiliations:** 1 Sultan Qaboos University

This review has been migrated. The reviewer awarded 2 stars out of 5 This is a useful small paper detailing the experiences of introducing case-based teaching supported by LMS video materials in pre-clinical teaching. The student evaluation shows that the process was successful, and serves as a point from which to expand. There are, however a number of points that reduce the significance of the study beyond the immediate experience and location. • “For the qualitative data analysis, the thematic analysis undertaken in this study followed a grounded theory approach by inductively extracting themes from participant responses, and then developing these themes into theory by identifying the organizing concepts which linked themes.” I’m afraid that this is not Grounded Theory – this appears to be only extracting themes from qualitative data. A crucial component of Grounded Theory is iterative data gathering and theming until saturation; the data-gathering process and results from each participant or group determine the sample size. This process has not been followed in this study. In your study, the number of participants / sample size was pre-determined, data were gathered and then themed, irrespective of whether saturation had been reached.• When reporting statistics, it would be useful to report raw numbers in addition to percentages. • On your statistics, it would have been useful if you had applied some sort of statistical testing to look for statistically significant differences. For example, 44% versus 37% appears to be considered different, yet TT = 42%, BIL = 38% appears to be considered “roughly similar”. Yes, the difference in the second pair of figures is smaller than the difference of in the first, but there is no explanation for a cut-off point which appears to be arbitrary.• “The literature suggests this is the first study to have evaluated the effectiveness of a blended and integrated learning approach in pre-clinical undergraduate medical education.” “Another limitation is that the described BIL approach does not have a precedent in Medicine, and while based on a sound educational theory, all the resources were developed de novo by staff of the JCU medical school.” o No educational theory was given in the paper, so it is unclear which educational theory forms the basis of this study.o I would suggest that, unless the researchers are using this terminology in an extremely narrow and specific sense, the fault lies with their literature search. Case-based and integrated pre-clinical teaching using materials (documents, audio and video) in an LMS has been going on for at least two decades, and has quite a bit of literature on the topic. In fact, much of the literature has moved on beyond the activities described in this study. (Actually, many Australian universities have been pace-setters in this field, so the researchers will be able to find rich expertise on their own door-step). More reference to the teaching methodology is given below.• On the teaching of the class:o From the description of the videos, it appears that they were merely a recording of a standard lecture, with some quiz questions interspersed. While this is a start, I would like to suggest that, if the researchers wish to really use the media available to them, they consider this study a small pilot only, and expand their repertoire to include other and more engaging methods of education. o One of your suggested improvements is the supplying of the answers to the quizzes. The danger of that is that students will merely breeze through the quiz and then learn the answer that has been given to them. Rather, if the student’s answer is correct, this can be confirmed; if, however, the student’s answer is incorrect, the correct answer should not be shown: rather explain why the selected answer is incorrect, perhaps give a hint, and let the student attempt the question again.So, while the study is a useful start, especially for their experience and the experience of their students, I would suggest that the researchers become far more familiar with ground work (and theory) that has already been done in the area, and then build upon that.

Reviewer Expertise:

NA

No decision status is available
